# Knowledge Regarding Organ Donation and Willingness to Donate among Health Workers in South-West Nigeria

**Published:** 2016-02-01

**Authors:** R. Oluyombo, M. B. Fawale, R. W. Ojewola, O. A. Busari, O. J. Ogunmola, T. O. Olanrewaju, C. A. Akinleye, Y. O. Oladosu, M. A. Olamoyegun, B. A. Gbadegesin, O. O. Obajolowo, M. O. Soje, A. Adelaja, L. M. Ayodele, O. E. Ayodele

**Affiliations:** 1 Renal Unit, Internal Medicine Department, Federal Teaching Hospital, Ido-Ekiti, Ekiti State, Nigeria,; 2 Neurology Unit, Department of Internal Medicine, Obafemi Awolowo University Teaching Hospital Ile-Ife, Osun State, Nigeria,; 3 Urological Surgery Unit, Department of Surgery, Lagos University Teaching Hospital, Idi-Araba, Lagos, Nigeria,; 4 Cardiology Unit, Department of Internal Medicine, Federal Teaching Hospital, Ido-Ekiti, Ekiti State, Nigeria,; 5 Renal Unit, Medicine Department, University of Ilorin Teaching Hospital, Ilorin, Kwara State, Nigeria,; 6 Department of Community Medicine, Ladoke Akintola University of Technology Teaching Hospital, Osogbo, Osun State, Nigeria,; 7 Internal Medicine Department, Ladoke Akintola University of Technology Teaching Hospital, Ogbomoso, Oyo state, Nigeria,; 8 Renal Unit, Department of Internal Medicine, Ladoke Akintola University Teaching Hospital, Ogbomoso, Oyo State, Nigeria,; 9 Renal Unit, Ladoke Akintola University of Technology Teaching Hospital, Osogbo, Osun State, Nigeria,; 10 *Mental Health Unit, Federal Teaching Hospital, Ido-Ekiti, Ekiti State, Nigeria*

**Keywords:** Organ transplantation, Health Knowledge, attitudes, practice, Nigeria, Community health workers, Health personnel

## Abstract

**Background:**

Organ transplantation program in developing countries is still significantly dwarfed. Health workers are undeniably important in the success of transplantation.

**Objective:**

To assess the knowledge and attitude of health workers toward organ donation in South-West Nigeria with a view to explaining reasons for these shortcomings.

**Methods:**

In a cross-sectional study conducted on 850 health care workers, self-administered questionnaires were used to obtain information from participants.

**Results:**

Of 850 participants, 766 (90.1%) returned their completed questionnaires. The mean±SD age of participants was 36.7±9.2 years. Majority (93.3%) of participants had heard of organ donation; 82.5% had desirable knowledge. Only 29.5% and 39.4% would be willing to donate and counsel potential organ donors, respectively; 36.5% would consider signing organ donation cards. Only 19.4% believed that organ transplantation is often effective and 63.4% believed they were permitted by their religion to donate. Permission by religion (OR 3.5; 95% CI 2.3 to 5.3), good knowledge (OR 2.9; 95% CI 1.4 to 5.7), readiness to sign donation cards (OR 2.6; 95% CI 1.7 to 3.8), discuss organ donation (OR 2.7; 95%CI 8.0 to 63.8), and knowing somebody who had donated (OR 2.9) independently influenced willingness to donate organ.

**Conclusion:**

There is disparity in knowledge of organ donation and willingness to donate among health care workers. Efforts should be intensified to give comprehensive and appropriate education to health care workers about organ donation to bridge this gap.

## INTRODUCTION

The burden of chronic diseases, such as chronic kidney disease (CKD), leading to organ failure is high and keeps increasing. The loss to organ failure worldwide is alarming and the available and effective treatment of end-stage organ failure such as CKD is transplantation. Prevalence of CKD from community studies in Nigeria ranges between 16% and 26% [1-2], and 6.7% to 8.4% [3-4] constituted medical admissions. Transplantation is not only cost-effective but also saves and improves quality of life [5, 6]. The cost of the alternative treatment for organ failure such as dialysis in kidney failure and outcome is both compelling and poor [7, 8]. However, the gap between supply and demand for organ donation widens daily and needs continuous activities and program to fill. 

Thirteen years after the first transplantation in Nigeria, organ transplantation is still being significantly dwarfed by non-availability of donors as a major factor [5]. Transplantation in Nigeria is limited, as in most other African countries, to living donors. Demand for organs grows as prevalence for chronic diseases such as hypertension [9], diabetes [11, 12], and daunting burden of infectious diseases, which contribute to organ failure in the region [13], increases. There is no national coordination of the program with transplantation activities coordinated by each center based on their local protocol. The process of funding of transplantation is majorly by patients and their families with just 10.5% of government support for the few [5]. There is no health insurance coverage for either treatment of end-stage kidney failure or organ transplantation. Inferentially, even though Nigerian constitution does not forbid organ donation, there is no enabling legislative framework to guide the professionals or protect the donors or relations. In spite of this major challenge, the professionals have undoubtedly demonstrated great skills through collaboration among centers and the outcome of kidney transplantation, for instance, is comparable with developed world [5].

The third WHO Global Consultation on Organ Donation and Transplantation advocates self sufficiency [14]. Striving to achieve self sufficiency optimizes the resources available within a country to meet demand for organ donation and transplantation. Each country and region needs to activate all opportunities to increase the yield of organs for transplantation. Attitudes and lack of knowledge among health care workers (HCWs) have been identified as barrier and pivotal to successful organ donation [15]. 

In view of the perennial shortage of organs for donation in spite of increasing transplant centers in the country, we hypothesized that HCWs who are crucial to successful transplantation are not favorably disposed to organ donation. We looked at the knowledge and belief of HCWs toward organ donation in South-West Nigeria. 

## MATERIALS AND METHODS

This is a cross-sectional study involving two tertiary health institutions, two secondary health institutions (general hospitals) and four primary health centers in Osun and Ekiti states of Nigeria. The hospitals and health centers were non-transplant centers but in the radius of about 80–100 km to transplant centers. The study was carried out to mark World Kidney day 2012. The period of the study was between January 2012 and June 2012.

The study was conducted using a self-administered questionnaire. The questionnaire had three parts, which seek information on socio-demography, knowledge, and attitude of the participants toward organ donation. Doctors were excluded from the study. Except the time when the questionnaire was rejected, consecutive health care workers were given the questionnaire.

The participants’ income was classified arbitrarily as “low,” “medium,” and “high” based on the monthly income of US$ ≤300, 301–500, and >500, respectively. Level of education was considered “medium” and “high” in HCWs with up to secondary school, and higher than secondary school education, respectively. Knowledge of the respondents was assessed as “poor,” “fair,” or “good” (desirable) through scores from questions answered correctly regarding meaning and awareness of the terms “organ donation,” “risks and effectiveness of organ donation,” “legislation and consents in organ donation,” and “the sources of information for their knowledge.” Other questions were on readiness to donate, influence of religion, who the participants would like to donate organ to, the most important factor to consider before donating such organs, whether they have seen anybody who donated, whether they would discuss organ donation with the family of a potential donor, signing of organ donation card if available, and whether their own family will allow their organ to be donated. The study was approved by the Research and Ethical Committees of the hospitals.

Statistical Analysis

Data was analyzed with SPSS^®^ for Windows^®^ ver 20. Participants who have never heard of organ donation were excluded from the main analysis. χ^2^ was used for the analysis of proportions of categorical variables. Independent-sample *Student’s t* test was used to compare means for two groups of variables. Logistic regression analysis of factors affecting willingness to donate using various variables with significance levels at p=0.02 from univariate analysis was used to determine the independent predictors of organ donation. A p value <0.05 was considered statistically significant.

## RESULTS

Out of 850 questionnaires distributed, 766 were returned giving a response rate of 90.1%. The mean±SD age of the participants was 36.7±9.2 years; 88.6% of participants were female who were younger than male counterparts (41.6±9.0 *vs* 32.4±7.2, p=0.001); 629 (82.1%) of the participants were Christians (Table 1). Of 715 (93.3%) who have heard of organ donation, only 88 (12.3%) would definitely want to donate while 45% would want to think about it.

**Table 1:** Socio-demographic characteristics of participants and levels of health care

Characteristics	Male n (%)	Female n (%)	Total n (%)	p value
Educational level				
Medium	4 (5)	59 (8.7)	50 (7.0)	
High	83 (95)	620 (91.3)	665 (93.0)	0.191
Religion				
Christianity	57 (66)	572 (84.2)	629 (82.1)	
Islam	30 (35)	107 (15.8)	137 (17.9)	0.001
Marital status				
Single	15 (17)	93 (13.7)	108 (14.1)	
Married	72 (83)	586 (86.3)	658 (85.9)	0.371
Monthly income				
Medium	10 (16)	197 (30.3)	207 (29.0)	
High	54 (84)	454 (69.7)	508 (71.0)	0.014

Knowledge and Beliefs of HCWs about Organ Donation

HCWs participated in this study heard about organ donation from doctors (13.7%), newspapers only (8.7%), and combination of other sources like television and radio (67.4%). Scoring the knowledge about organ donation, 590 (82.5%) were assessed to have desirable knowledge. Four-hundred and fifty-three (63.4%) participants believed that their religion allows organ donation and 494 (69.1%) would prefer to donate to only their family members. Only 2 (0.3%) would consider religion of recipients while 33.3% and 19.2% considered relation to and age of the prospective recipient important factors for donation. For living donation, 430 (60.1%) believed donor should give consent while 340 (47.6%) and 135 (18.9%) of HCWs believed family and spouse should give consent for cadaveric organ donation, respectively. Five-hundred and four (70.5%) agreed that organ donation should be promoted.

Only 139 (19.4%) believed that organ transplantation is often effective; 541 (76.2%) believed it is sometimes effective. More participants (574 [74.9%] *vs* 502 [65.5%]) believed that kidney transplantation is more effective than liver transplantation. Four-hundred and fifty (62.9%) got the correct definition of “organ donation” as “the removal of the tissues of human body for the purpose of transplantation to another person” and 662 (92.6%) agreed that organ donation is to save life. Five-hundred and fifty-nine (78.2%) believed organ donation is associated with risks and only 22 (3.1%), and 6 (0.8%) knew somebody who had donated and waited for transplantation, respectively.

About the existence of a local or an international legislation to regulate organ donation, 362 (50.6%) said “no such legislations exist;” 512 (71.6%) believed there is need for laws to govern organ donation. In answering “which organ could be donated?” 684 (89.3%) knew that kidney, heart, eyes, and liver could be donated. Majority (545 [75.6%]) believed that organ donated could be misused or misappropriated.

Being female (p=0.001), having higher education (p=0.035) and income (p=0.004) were positively associated with higher knowledge scores. Knowing the correct definition of organ donation and belief that transplantation is effective were all significantly associated with better knowledge scores (p<0.05). Similarly, HCWs who knew somebody who had donated organ (p=0.001) or waited for transplantation (p=0.001) also had high knowledge scores. 

Willingness of HCWs to Donate

Only 211 (29.5%) of studied HCWs were willing to donate; 218 (36.4%) would be willing to discuss organ donation when caring for a potential donor, and 202 (28.3%) would never discuss organ donation. More females (30.3%) than males (21.9%) would be willing to donate (p=0.160) (Table 2). People whose religion permits to donate, would also be more willing than those who believed their religions do not give them permission to donate. However, more males than females (68.8% *vs* 33.3%, p=0.001), and HCWs with higher education than others (64.2% *vs* 54.0%, p=0.148) would consider signing organ donation cards. Similarly, HCWs who had the permission of their religion to donate were more likely to consider signing organ donation card if asked to do so (p=0.001). If asked to sign organ donation card, only 261 (36.5%) would do so; the families of only 46 (6.4%) HCWs were likely to grant permission for organ donation at death; 386 (54%) families were unlikely to agree. 

**Table 2:** Factors affecting willingness of health care workers to donate organ

Factor	Willingness to donate		p value
	Yes, n(%)	No, n(%)	
Gender			
Male	14 (22)	50 (78)	0.160
Female	197 (30.3)	454 (69.7)	
Marital status			
Single	29 (28.2)	74 (71.8)	0.740
Married	182 (29.7)	430 (70.3)	
Level of education			
Up to secondary school	20 (40)	30 (60)	0.090
Diploma and above	191 (28.7)	474 (71.3)	
Religion allows organ donation?			
No	42 (16.0)	220 (84.0)	<0.001
Yes	169 (37.3)	284 (62.7)	
Monthly income			
Medium	56 (27.1)	151 (72.9)	0.350
High	155 (30.5)	353 (69.5)	
Signing of organ donation card?			
No	101 (22.2)	353 (77.8)	<0.001
Yes	110 (42.1)	151 (57.9)	
Discussing organ donation?			
No	42 (19.3)	176 (80.7)	<0.001
Yes	169 (34)	328 (66.0)	
Knowing anyone who had donated organ?			
No	192 (27.7)	501 (72.3)	<0.001
Yes	19 (86)	3 (14)	
Knowing somebody waiting for transplantation?			
No	205 (28.9)	504 (71.1)	0.001
Yes	6 (100)	0 (0)	
Knowledge of organ donation score			
Good	26 (20.8)	405 (68.6)	0.019
Fair	185 (31.4)	99 (79.2)	

Out of 211 participants who would be willing to donate 22.2% would be ready to sign organ donor card if asked to do so. Willingness to donate was higher but not significantly different among the three tiers of health care levels (42.7% *vs* 29.9% *vs* 27.5%, p=0.602). Table 3 shows the results of the multivariate logistic regression analysis. HCWs whose religion permits organ donate, were independently associated with willingness to donate (OR 3.79, 95% CI 2.51 to 5.76). Knowing somebody who had donated, readiness to sign organ donation card, and high knowledge score, independently influenced willingness to donate organ.

**Table 3:** Multivariate analysis of factors that influence willingness to donate at all levels of health care

Factors	Coefficient	OR (95% CI)
High education	0.245	1.29 (0.65 to 2.50)
Permission by religion	1.334	3.79 (2.51 to 5.76)
Can discuss organ donation with relations	1.536	4.65 (2.82 to 7.66)
Know a previous donor	2.119	8.32 (2.19 to 31.70)
Would sign organ donor card	1.123	3.07 (2.07 to 4.56)
Good knowledge score	0.016	1.02 (0.58 to 1.78)

## DISCUSSION

We found that only 29.5% of studied HCWs were willing to donate organ, though there was high level of awareness (93%) and knowledge (82.5%) about organ donation. There were varying reports of willingness to donate organ among HCWs in Nigeria. Anochie, *et al* [15], reported that <50% of medical students studied would be willing to donate kidneys for transplantation. Aghanwa, *et al* [16], in Ile-Ife and Agaba, *et al *[17], in Jos reported willingness to donate kidneys in 52.1%, and 75.6% of HCWs, respectively. These two studies were conducted for living kidney donation in teaching hospitals with established renal treatment centers and medical doctors were included while our study considered organ donation—both living and cadaveric—excluding doctors, among HCWs in the three tiers of health care. In addition, our study was conducted in the southwestern part of the country with predominantly Yoruba tribe. LLiyasu, *et al* [18], reported low willingness to donate among this cohort compared to other tribes in a study conducted in the northern part of the country.

Only 1 in 4 of those who were willing to donate was ready to sign donor card. In Germany, only 1 in 10 of people in support of organ donation signed donor card [19], and one-fourth of adults in the UK [20], have registered on the national donor registry. In another study, among HCWs in Turkey, 44.2% were willing to donate but only 17.9% carried donor cards [21]. It is a situation of inability to action good intention. Plausible reasons are socio-cultural beliefs and traditions. Agreeing to sign organ donation cards imply mutilation of body at death or after, which according to many is a taboo and against religious belief.

Almost half of the HCWs studied were not decisive. This implies needs for activities that motivate and encourage positive attitude through appropriate information dissemination. Within 13 years, Portugal reported over 100% increases in organ donation by training health professionals [7]. Acquaintance with transplantation process in established centers should form part of the training because less than 1% and 5% of HCWs have known someone who had donated and waited for transplantation, respectively. 

The proportion of participants who would be ready to discuss organ donation with relations of potential patients for cadaveric donation was low but associated with positive attitude toward donation. The main reasons for this deficiency might be because of inexperience and inadequate information. Medical staffs are the first to establish contacts with potential donors and if they are well-informed and convinced, could engender successful organ donation [22]—both living and cadaveric. 

As much as two-thirds of studied HCWs agreed that organ donation should be promoted even though they were aware that it is not without risks. This may be for the fact that it saves life as expressed by about two-thirds of HCWs. Meanwhile, half of the participants knew there was no local law guiding organ donation with appropriate legislation in place. Various policies such as presumed consent (opt-out), enforced presumed consent and informed consent (opt-in) are in operation in Europe and other developed countries with established organ transplantation program. Framework of the establishment and national policy influence organ donation rate. Opt-out policy for instance, has been shown to increase organ donation rates in Spain [23, 24]. It is not impossible that socio-cultural complexities would be a factor in a country like Nigeria, as it was reported in other countries like Iran. Appropriate and considerate national policy on organ donation would ethically propel organ donation. It will also guide and protect both the service providers and users. 

Considering the high proportion of HCWs who believed organ donation was not without risks, it is important to educate the HCWs on the possible risks to donors of organ. Any seemingly gray areas should be tackled and wrong notion about risks should be demystified. However, with improvement in surgical skills in harvesting organs, peri-operative planning and appropriate legislation in the selection of donors, risk to donors have been minimized significantly [25]. Desirable experience of self-fulfillment and invaluable psychological relief as reported by Schnitzler, *et al* [26, 27], should justify sustainability of organ donation. Follow-up studies have shown good encouragement in short- and long-term safety of living kidney donation and morbidity and mortality remain low in organ donation [28-30]. 

Being female, having higher education, earning higher income and believing in the effectiveness of organ transplantation positively promote desirable knowledge of organ donation. This is similar to the findings of other study groups [31-33]. However, neither the level of education nor income significantly influenced willingness to donate in this study. Nina and Irwin reported that people with higher level of education tend to have skeptical attitudes toward organ donation [34]. More specific education directed toward organ donation rather than general education is more relevant in improving attitude of HCWs to organ donation [35, 36]. 

Many studies have emphasized the importance of religion [37, 38]. A report from the northern part of Nigeria revealed religion as an independent factor affecting willingness to donate [18]. Nigeria, even though constitutionally secular, has diverse religious affiliations. Therefore, to increase organ yield, it is imperative to include leaders of religion. No religion formally forbids principles of organ donation as a way to save life [39-41], but many controversies exist. Religion plays a pivotal role and was reported to have a significant influence among the Asians [42]. In Iran, Dehghani, *et al* [43], reported high level of family refusal to allow their relations to donate as it was difficult to accept the concept of brain damage because miracles could still happen. Lack of adequate information on the position of religion on organ donation could limit its invaluable benefits. Religion is a reality in human experience that finds expression in beliefs, relationship and practice. For effective and successful transplantation program, health authority should organize forum for constructive debates for clerics of various religion as they have great influence on the worshippers. 

In conclusion, this study showed that majority of HCWs would want organ donation promoted and were aware of risks. We also observed a wide margin between knowledge of organ donation and willingness to donate. However, the factors recognized among HCWs in this study are amenable to change. A pragmatic and comprehensive medical education, adequate information to improve knowledge and exposure coupled with improved religious knowledge that demystifies area of concern, fear and confusion is warranted. We also believe that this study would form a template for full-fledged organ donation—including cadaveric transplantation in the country as this is not farther from now again.

## Notes

**FUNDING SUPPORT:** None.
